# Multidisciplinary Management of Lymphangioleiomyomatosis Presenting With Severe Bilateral Pneumothorax During Pregnancy: A Case Report

**DOI:** 10.1155/crpu/5512688

**Published:** 2026-08-31

**Authors:** Shiva Ebrahimi, Ali Abdollahzadeh, Maryam Barghaman, Amirhossein Hajialigol

**Affiliations:** ^1^ Department of Internal Medicine, Tabriz University of Medical Sciences, Islamic Azad University, Tabriz, Iran, tiau.ac.ir; ^2^ School of Medicine, Tabriz University of Medical Sciences, Tabriz, Iran, tbzmed.ac.ir; ^3^ Alborz Office of Universal Scientific Education and Research Network (USERN), Alborz University of Medical Sciences, Karaj, Iran, abzums.ac.ir

## Abstract

**Introduction:**

Lymphangioleiomyomatosis (LAM) is a rare, progressive, multisystem disease primarily affecting women of reproductive age. It is characterized by the abnormal proliferation of smooth muscle‐like LAM cells that infiltrate the lungs, lymphatics, and kidneys. This case highlights that the clinical value lies not in isolated features, but in the convergence of early gestational presentation, severe bilateral disease, diagnostic limitations, and the need for coordinated multidisciplinary decision‐making in a resource‐constrained setting.

**Case Presentation:**

A 23‐year‐old pregnant woman (G3P2L1A1) at 17 weeks of gestation presented with severe shortness of breath and bilateral shoulder pain, escalating to respiratory distress (SpO2 80%) and functional class IV within hours. Imaging revealed bilateral pneumothorax, near‐complete lung collapse, and multiple small cysts in the lung parenchyma, suggestive of LAM. Chest tube placement and pulmonary consultations were performed, followed by a control CT scan confirming LAM. Because of the risk of worsening LAM during pregnancy, termination of the pregnancy was carried out, and diagnostic confirmation was achieved through video‐assisted thoracoscopic surgery (VATS) with lung biopsy, showing cystic and adhesive lesions. Treatment included decortication, lesion removal, and sirolimus therapy, an mTOR inhibitor. The patient was discharged in stable condition with ongoing medical follow‐up.

**Conclusion:**

Management of LAM during pregnancy requires a multidisciplinary approach, emphasizing personalized care to balance maternal and fetal health. This case highlights the need for vigilance and adaptability in the care of pregnant women with rare pulmonary disorders like LAM. Early diagnosis is key to slowing the progressive decline of pulmonary function. Further research is needed to improve treatment options for LAM patients.

## 1. Introduction

Lymphangioleiomyomatosis (LAM) is an uncommon progressive disorder that predominantly affects women of reproductive age and may involve the lungs, lymphatic system, and kidneys. The disease is driven by proliferation of abnormal smooth muscle‐like LAM cells, which gradually disrupt normal lung architecture and contribute to cyst formation, airflow limitation, lymphatic abnormalities, and in advanced cases, respiratory failure [1, 2]. Clinically, LAM may remain unrecognized until patients develop exertional dyspnea, chest pain, cough, or spontaneous pneumothorax, making early diagnosis challenging. Pulmonary involvement is central to the clinical course of LAM. Although chest radiography may show nonspecific findings such as increased lung volume, reticular or cystic‐appearing changes, or pneumothorax, plain radiographs may underestimate the extent of disease. Pneumothorax is particularly important because it may be the first major clinical event and can recur during the disease course [3]. In pregnant patients, this complication carries additional risk because maternal respiratory compromise can rapidly affect both maternal stability and fetal viability.

High‐resolution computed tomography (HRCT) is the key imaging modality for identifying the characteristic cystic lung pattern of LAM. The typical appearance consists of multiple thin‐walled, round cysts distributed throughout otherwise relatively preserved lung parenchyma. Cyst size and burden vary between patients and may increase with disease progression, although imaging interpretation can be difficult when extensive pneumothorax or lung collapse limits full parenchymal assessment [3, 4]. These imaging findings are highly suggestive but should be interpreted together with clinical context and other diagnostic evidence. Diagnosis can often be supported by characteristic HRCT findings, extrapulmonary manifestations, and serum biomarkers such as vascular endothelial growth factor‐D (VEGF‐D), where available. However, tissue confirmation remains important when noninvasive findings are insufficient or biomarker testing is unavailable. Histopathological examination typically demonstrates cystic architectural change with proliferation of spindle or epithelioid LAM cells, and immunohistochemical staining may support the diagnosis through expression of melanocytic and smooth muscle markers such as HMB‐45 and desmin [5].

We report a case of LAM in a 23‐year‐old pregnant woman who presented at 17 weeks of gestation with severe bilateral pneumothorax, near‐complete lung collapse, and acute respiratory compromise. The diagnosis was confirmed by thoracoscopic lung biopsy and immunohistochemical staining. This case highlights the clinical difficulty of managing severe LAM during pregnancy, particularly when rapid multidisciplinary decision‐making is required in a setting with limited access to noninvasive diagnostic biomarkers.

## 2. Case Presentation

A 23‐year‐old woman (G3P2L1A1) at 17 weeks of gestation was admitted to the hospital with a chief complaint of shortness of breath and pain in both shoulders that began the day prior to admission. Within a few hours, the patient′s shortness of breath intensified, reaching functional class IV (FCIV). Upon arrival at the city′s medical center, at presentation, the patient demonstrated multiple indicators of life‐threatening maternal compromise, including severe hypoxemia (SpO₂ 80% on admission), respiratory distress with gasping, FCIV dyspnea, and respiratory acidosis on arterial blood gas analysis. Imaging revealed bilateral pneumothorax with near‐complete lung collapse, further compromising effective ventilation. Despite initial chest tube placement, the patient remained clinically unstable with persistent respiratory insufficiency. These findings indicated a high risk of rapid clinical deterioration and potential respiratory failure requiring invasive ventilatory support. Pregnancy and high estrogen levels can exacerbate LAM, promoting cell proliferation and worsening symptoms. Management includes supportive care, such as smoking cessation and avoiding air travel, and pharmacologic interventions like sirolimus, an mTOR inhibitor that helps to stabilize lung function. After receiving appropriate treatment, the patient was discharged from the hospital in good general condition and was treated as medical follow‐up with sirolimus 2 mg daily. (Table 1).

**Table 1:** Timeline of clinical course, diagnostic workup, and management.

Time point	Clinical events and findings	Interventions/decisions
Day 0 (Admission)	23‐year‐old pregnant woman (17 weeks gestation) presented with acute dyspnea, bilateral shoulder pain, SpO₂ 80%, respiratory distress (FC IV)	Initial stabilization; transfer to tertiary center
Day 0 (emergency evaluation)	Spiral CT scan revealed severe bilateral pneumothorax with near‐complete lung collapse	Bilateral chest tube insertion
Day 1–2	Persistent respiratory instability; ABG showed respiratory acidosis	Multidisciplinary evaluation (pulmonology, thoracic surgery, obstetrics/gynecology)
Day 2–3	HRCT demonstrated diffuse small cysts (< 1 mm) suggestive of LAM	Diagnostic consideration of LAM; further planning
Day 3–5	Ongoing maternal risk due to severe respiratory compromise at 17 weeks gestation	Decision for pregnancy termination following multidisciplinary discussion and consent
Poststabilization	Clinical improvement after initial management	Proceeded to VATS for definitive diagnosis
Post‐VATS	Intraoperative findings: cystic lesions and adhesions; biopsy obtained	Decortication and wedge biopsy performed
Pathology	Histopathology and immunohistochemistry positive for HMB‐45 and desmin	Definitive diagnosis of LAM confirmed
Postoperative period	Clinical stabilization and recovery	Initiation of sirolimus therapy (2 mg/day)
Follow‐up	Improved functional status; stable clinical condition	Ongoing outpatient follow‐up with planned imaging and pulmonary assessment

### 2.1. Diagnostic Findings

**Figure 1:**
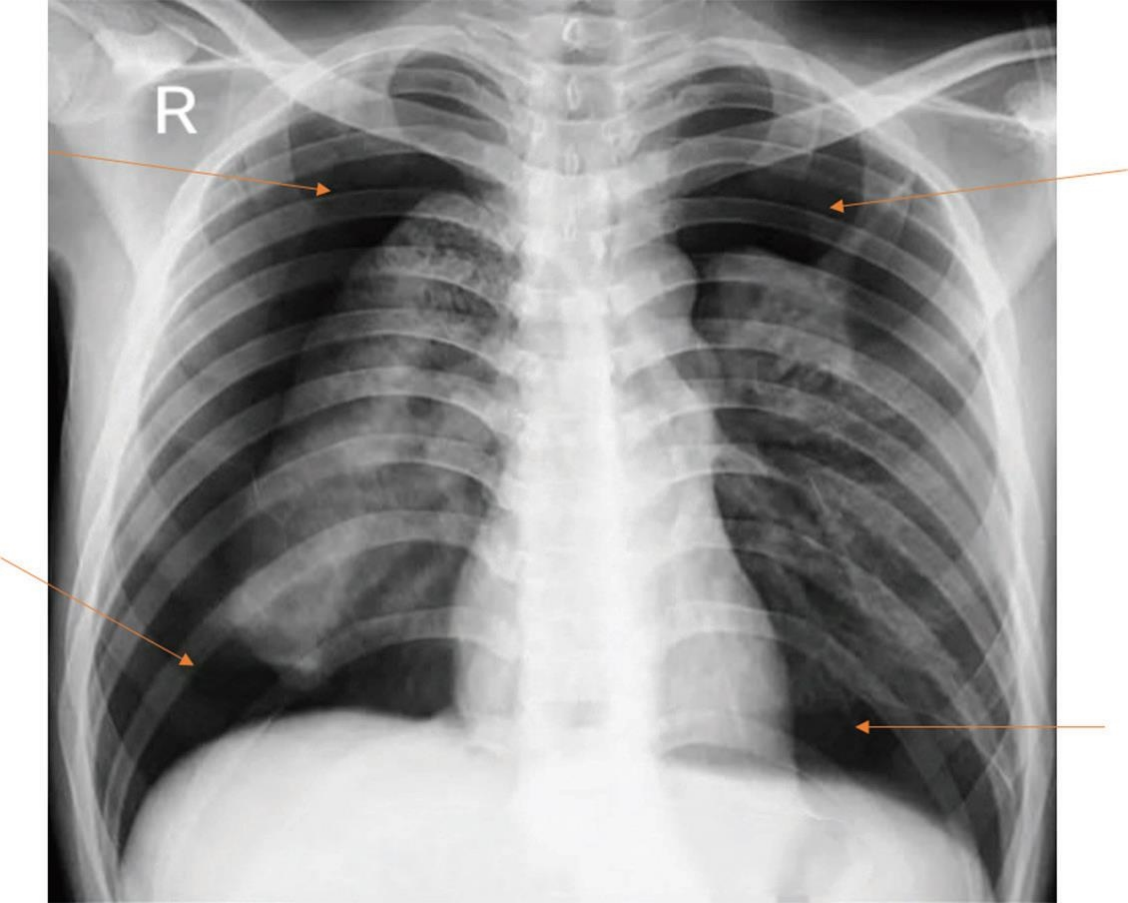
CXR: Before placement of chest tube (arrows showing pneumothorax).

**Figure 2:**
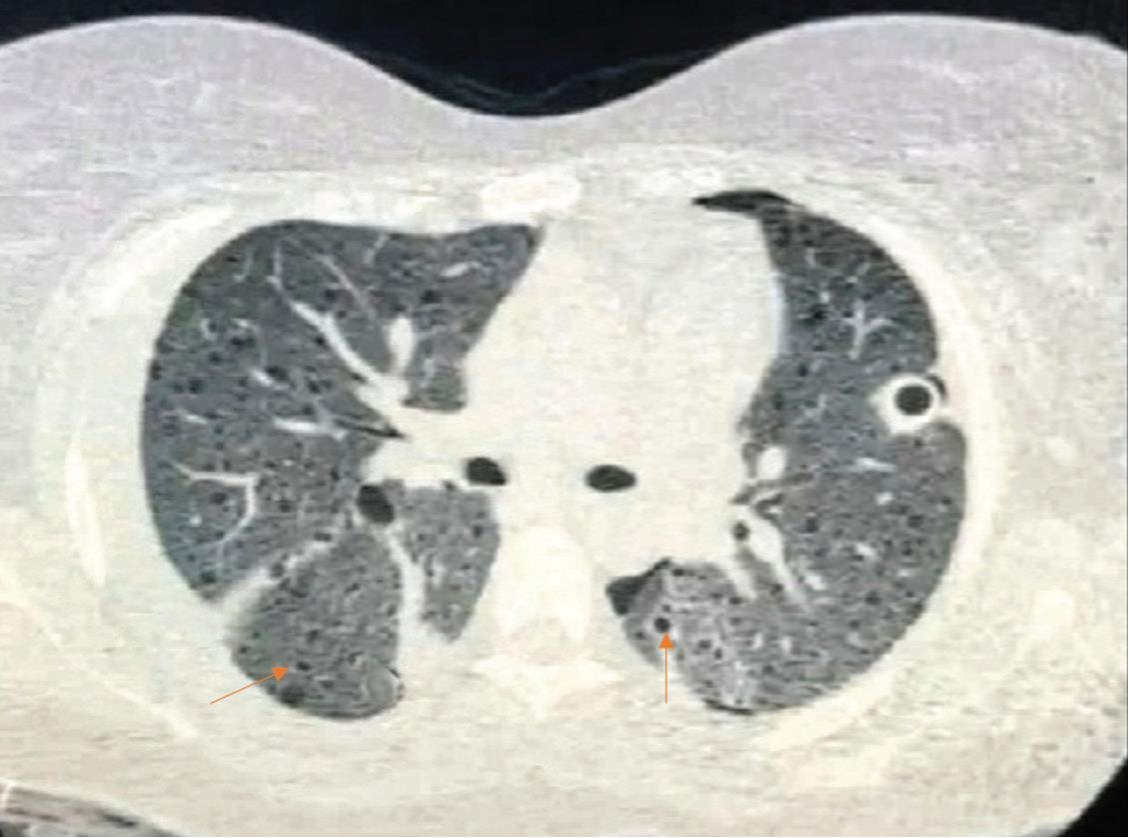
CT scan after placement of chest tube (arrows showing the cysts).

Multiple small cysts are observed in the parenchyma of both lungs. The findings may suggest LAM. No significant pleural effusion or pericardial effusion is detected. (Figure 1) She underwent chest tube placement by the surgical service and a pulmonary consultation has been requested for the patient. In the primary CT scan, very small cysts with a size of less than 1 mm were seen in the parenchyma of both lungs, but because of the large size of the pneumothorax, it was not possible to see the parenchyma completely, so a control CT scan (HRCT) was performed, which demonstrated diffusely distributed, small, thin‐walled cystic lucencies throughout both lungs, which were suggestive but not diagnostic of LAM. Given the extensive pneumothorax and partial lung collapse on initial imaging, precise measurement of cyst size was not feasible, and previously reported dimensions should be interpreted as approximate rather than exact. The cysts appeared round and were surrounded by relatively preserved lung parenchyma without dependent predominance, which helped distinguish them from atelectasis or collapse‐related changes. These radiologic features supported a cystic lung disease pattern and prompted further diagnostic evaluation. The cystic changes were diffusely distributed without lobar predominance and were characterized by round, thin‐walled lucencies, supporting a cystic lung disease pattern rather than dependent atelectasis or parenchymal collapse. The patient was extubated after a few days, and because of the possibility of LAM worsening during pregnancy, gynecological and forensic consultations were performed, and the pregnancy was terminated by curettage. (Figure 2).

A lung biopsy and video‐assisted thoracoscopic surgery (VATS) were performed to confirm the diagnosis. Spirometry could not be performed at initial presentation because of the patient′s critical respiratory status. Longitudinal pulmonary function testing was not consistently available during follow‐up because of limited access to pulmonary function laboratory services.

#### 2.1.1. VATS

##### 2.1.1.1. Decortication + Wedge‐Shaped Lower and Middle Lobe Biopsy

**Figure 3:**
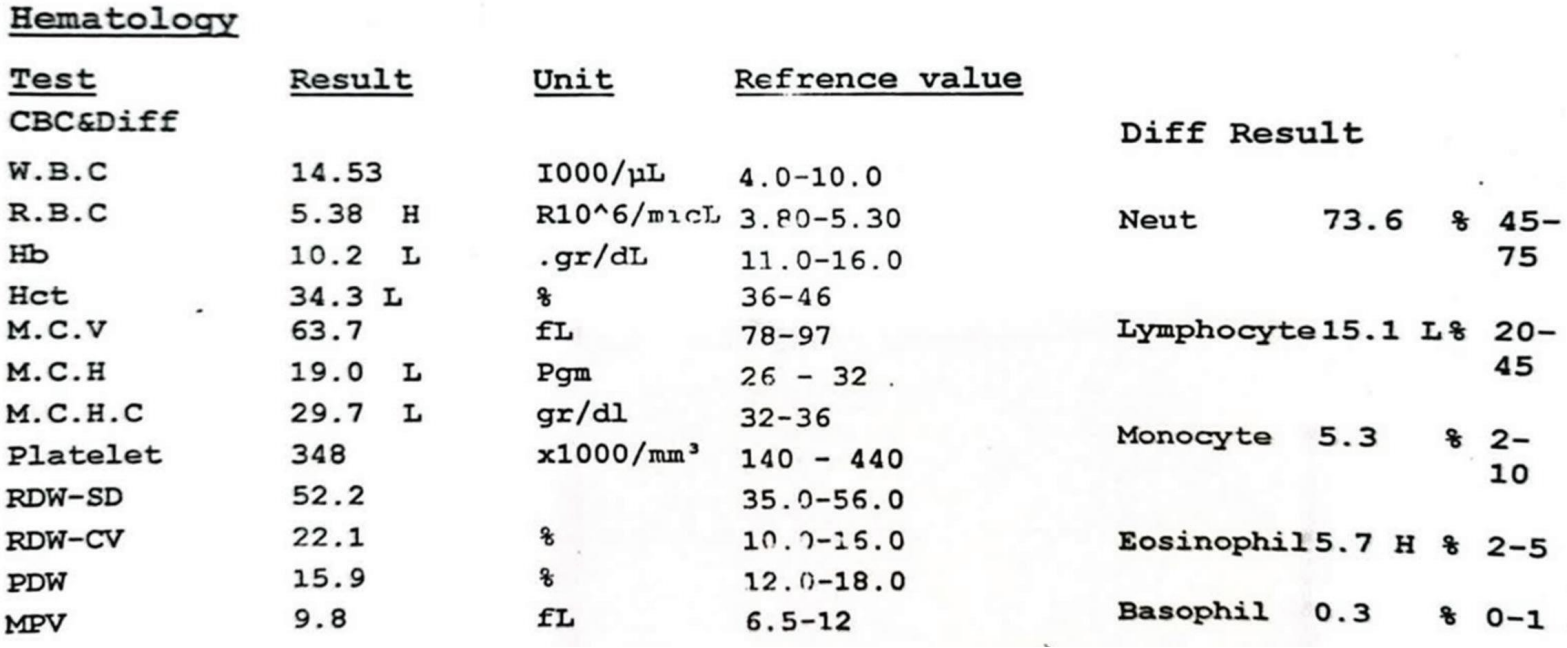
Lab data.

In the sterile operating room conditions, the patient was placed in the side up position and under general anesthesia, and a 10‐gauge crocar was inserted in the fourth and fifth intercostal spaces. Two other 10‐gauge crocars were inserted posteriorly and one space above and below. In direct visualization of the right lung, gelatinous adhesions were seen all around the lung and the lung was collapsed. Cystic lesions were seen in all three lobes of the lung. The adhesions and cysts that were seen were released and the gelatinous adhesions were suctioned and removed. The patient was placed under manual ventilation, and the lung was fully expanded and two chest tubes were inserted. Labratory tests are shown in Figure 3.

**Figure 4:**
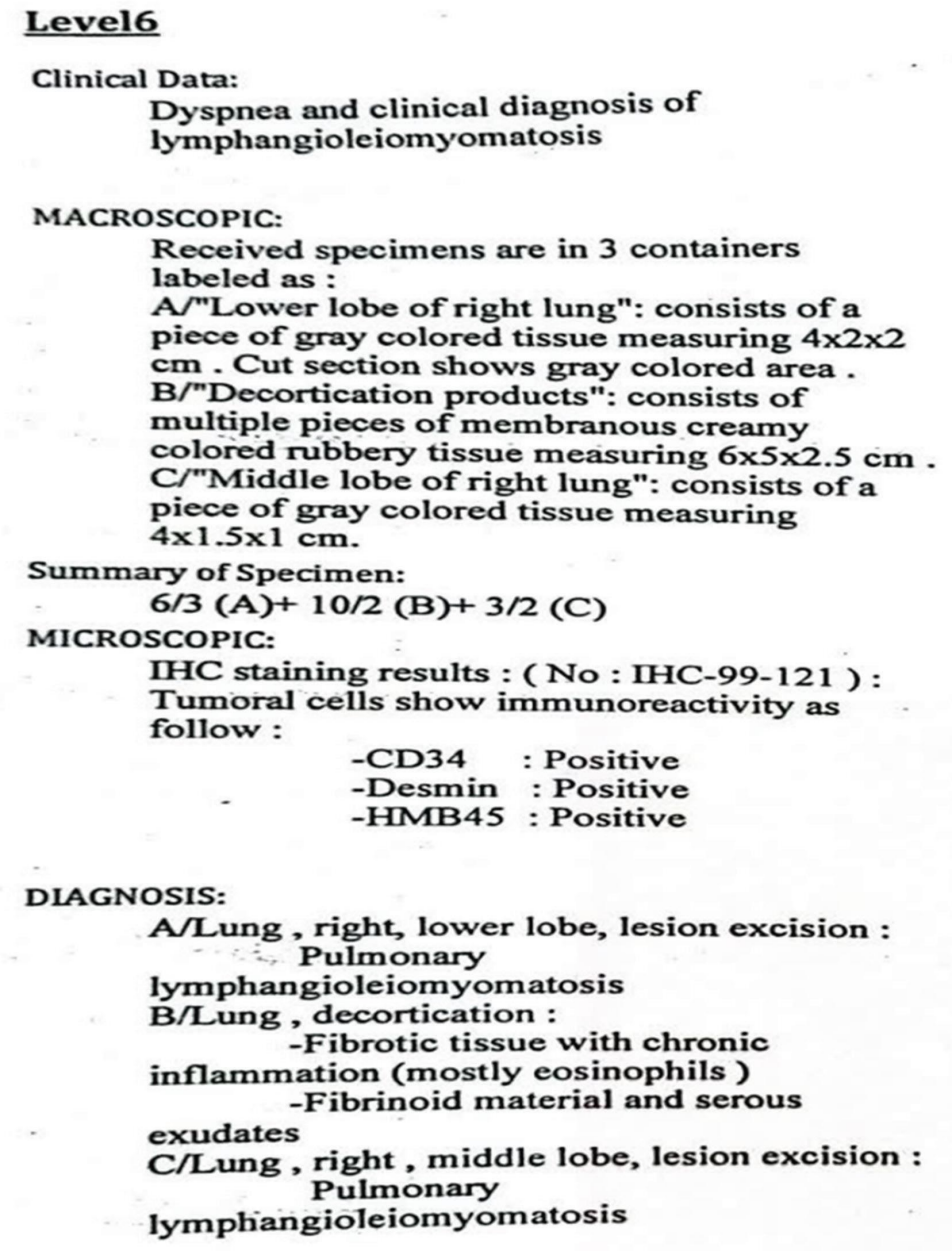
Pathology.

Pathological examination of the lung tissue often reveals cystic changes and a proliferation of spindle or epithelioid cells. Immunohistochemical staining demonstrated that the lesional cells were positive for HMB‐45 and desmin. CD34 highlighted vascular endothelial structures and was not expressed in the lesional cells. Histopathological examination revealed cystic architectural changes associated with a proliferation of spindle to epithelioid cells distributed along the cyst walls and interstitium. Immunohistochemical staining demonstrated positivity for HMB‐45 and desmin in the lesional cells, a pattern characteristic of LAM and supportive of perivascular epithelioid cell differentiation. CD34 highlighted vascular endothelial structures and was not expressed in the lesional cells. Smooth muscle actin (SMA) staining was not available in this case due to local laboratory limitations; however, the combined morphologic features and immunophenotype were considered sufficient for diagnosis. The staining pattern was interpreted in conjunction with histologic architecture and clinical‐radiologic findings, supporting a diagnosis of LAM rather than a nonspecific cystic lung disease. (Figure 4) Autoimmune serologic tests were conducted to exclude differential diagnoses, such as connective tissue diseases or autoimmune‐associated interstitial lung diseases (ILDs), which may present with cystic changes. An important differential diagnosis is Tuberous Sclerosis Complex (TSC)‐associated. TSC is a multisystem genetic disorder characterized by hamartomatous lesions affecting the skin, brain, kidneys, and lungs, and may be associated with pulmonary LAM [6]. However, in the present case, there were no clinical features suggestive of TSC, such as facial angiofibromas, hypomelanotic macules, seizures, or renal manifestations. The absence of these systemic findings supports a diagnosis of sporadic LAM rather than TSC‐associated disease.

### 2.2. Lab Tests

**Figure 5:**
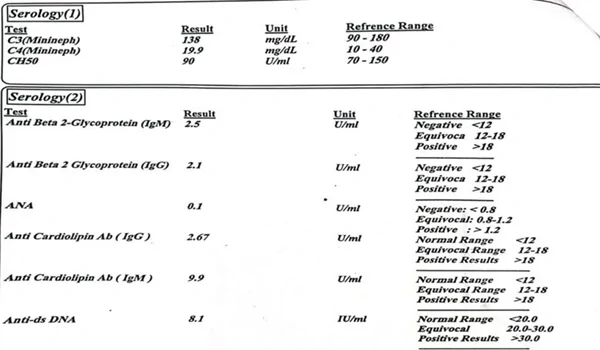
Serology.

Workups for LAM can include testing for anti beta 2 glycoprotein (IgM and IgG), ANA, anticardiolipin Ab (IgM and IgG) and anti‐dsDNA. All of them were within normal range (Figure 5).

**Figure 6:**
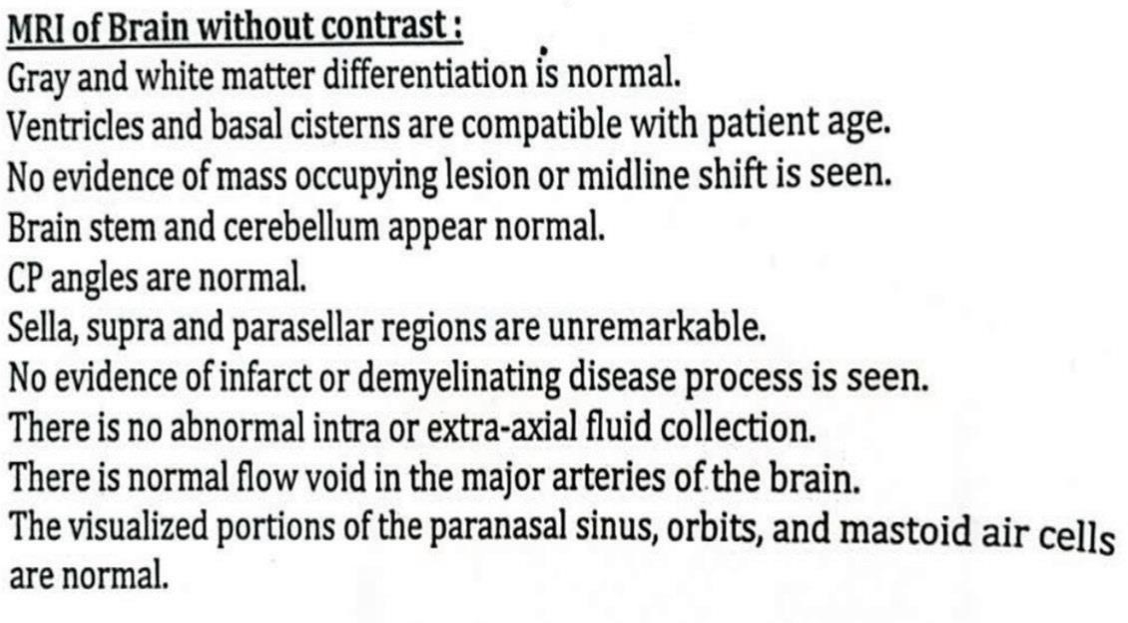
MRI report.

Whole‐body MRI was performed to evaluate for extrapulmonary manifestations of LAM and to assess for features suggestive of TSC, particularly renal angiomyolipomas. No abnormalities were identified, and there was no evidence of systemic involvement, supporting the diagnosis of sporadic LAM. (Figure 6).

### 2.3. Treatment and Management

Management decisions were made through a multidisciplinary approach involving pulmonology, thoracic surgery, and obstetrics/gynecology. At 17 weeks′ gestation, the patient presented with life‐threatening respiratory compromise, including severe bilateral pneumothorax and hypoxemic respiratory failure. Given the severity of maternal illness, continuation of pregnancy posed a significant risk to maternal survival. Alternative strategies, including expectant management with intensive monitoring and continuation of pregnancy until fetal viability, were considered. However, these options were deemed unsafe because of the patient′s unstable respiratory status and the need for urgent intervention. Early delivery was not feasible at this gestational age because of fetal nonviability. After multidisciplinary discussion and informed consent, the decision was made to proceed with pregnancy termination to prioritize maternal stabilization.

The patient underwent chest tube placement for the treatment of pneumothorax. Because of the possibility of LAM worsening during pregnancy, the pregnancy was terminated via curettage following gynecological and forensic consultations. Pregnancy can exacerbate LAM because of increased estrogen levels, which can promote LAM cell proliferation. After receiving appropriate treatment, the patient was discharged from the hospital in good general condition. After clinical stabilization, sirolimus was initiated at 2 mg/day, a dosing approach consistent with published LAM treatment experience and the standard‐dose regimen used in the MILES trial [7]. No documented dose adjustment was made during the reported follow‐up period. The patient underwent routine clinical follow‐up for treatment response and tolerance, including surveillance for common adverse effects of sirolimus: oral ulcers, gastrointestinal intolerance, acne, edema, infection, and respiratory symptoms. However, complete longitudinal data on serum trough levels and laboratory monitoring were not available for this report. Functional status improved from severe limitation (NYHA class IV) at presentation to independent ambulation without dyspnea during follow‐up. Her clinical course supports the established role of sirolimus in slowing disease progression and improving functional status in patients with LAM. The decision to terminate the pregnancy was based on a combination of critical maternal risk factors, including severe hypoxemic respiratory failure, bilateral pneumothorax with markedly reduced functional lung capacity, and ongoing clinical instability despite initial intervention. At 17 weeks′ gestation, fetal viability was not achievable, and continuation of pregnancy was expected to exacerbate maternal disease because of hormonal influences and increased physiological demands. The risk of further respiratory decompensation, need for prolonged mechanical ventilation, and potential maternal mortality were key considerations. Following multidisciplinary discussion involving pulmonology, thoracic surgery, and obstetrics/gynecology, and after obtaining informed consent, pregnancy termination was performed as a life‐saving intervention.

Given the known teratogenic potential of sirolimus and the risk of disease exacerbation during pregnancy, extensive counseling was provided regarding reproductive planning. The patient expressed no desire for future pregnancies, and long‐term management has focused on optimizing pulmonary stability and preventing recurrence of complications. She continues to undergo routine clinical follow‐up, including pulmonary function testing and periodic HRCT imaging, as part of comprehensive long‐term care for LAM.

## 3. Discussion

LAM is a well‐recognized, rare cystic lung disease primarily affecting women of reproductive age [8]. A hallmark of LAM is recurrent pneumothorax, which occurs in more than half of patients at some point during their disease course. As such, the presentation of pneumothorax in this patient, although dramatic because of bilateral involvement and pregnancy, is not an uncommon or novel manifestation [9]. The risk of pneumothorax recurrence in LAM is substantial, especially with advancing disease. Although pleurodesis is a recommended approach to reduce recurrence in many patients with LAM, it was not performed in this case during the initial episode [10, 11]. Pleurodesis was not performed during the index hospitalization because the patient presented with severe bilateral pneumothorax and profound respiratory instability, which made definitive pleural intervention unsafe in the acute phase.

Although individual features such as pneumothorax, pregnancy‐associated disease progression, and the need for surgical diagnosis are well recognized in LAM, the present case is distinguished by the combination of several high‐risk and context‐specific factors. These include presentation at an early gestational age, severe bilateral pneumothorax with near‐complete lung collapse, limited access to noninvasive diagnostic biomarkers, and the necessity for urgent multidisciplinary decision‐making. Therefore, the primary contribution of this report lies in illustrating how these factors intersect to influence clinical management, particularly in resource‐limited environments, rather than in any single novel clinical feature.

Current LAM guidelines recommend pleurodesis to prevent recurrence; however, this is typically undertaken once the patient is hemodynamically stable and lung expansion is adequate. In this case, the clinical priority was rapid stabilization, chest tube decompression, and evaluation of maternal–fetal risk. Pleurodesis will be reconsidered in future management to reduce the likelihood of recurrence. The severity of the patient′s condition was evident at presentation, characterized by FCIV dyspnea, profound hypoxemia (SpO₂ 80%), respiratory acidosis on arterial blood gas analysis, and bilateral pneumothorax with near‐total lung collapse on imaging. The combination of acute respiratory failure, extensive cystic involvement, and hemodynamic instability indicated a high‐severity clinical state requiring emergent multidisciplinary intervention. Counseling regarding future pregnancy was provided in accordance with current international recommendations. Pregnancy in LAM is considered high risk because of increased estrogen levels and the potential for worsening pneumothorax, chylous effusion, or accelerated decline in lung function. Pregnancy may be considered only in individuals with stable disease, preserved pulmonary function, and reliable access to specialized multidisciplinary care. As the patient did not desire future pregnancies and requires long‐term sirolimus therapy which is contraindicated during gestation she was advised to avoid conception and to use effective contraception. Pregnancy in patients with LAM is associated with an increased risk of disease progression and complications, including pneumothorax and respiratory deterioration. This is thought to be mediated by hormonal factors, particularly estrogen, which may promote LAM cell proliferation. Reported cases have demonstrated worsening respiratory symptoms and increased pneumothorax risk during pregnancy. In the present case, the patient′s life‐threatening respiratory condition at 17 weeks′ gestation precluded safe continuation of pregnancy or expectant management toward fetal viability. Therefore, termination was undertaken as a life‐saving intervention following multidisciplinary evaluation. In this case, pregnancy termination was undertaken in the context of clearly defined maternal risk indicators, including severe hypoxemia, bilateral pneumothorax with near‐total lung collapse, and persistent respiratory instability. These objective findings provided a strong clinical basis for prioritizing maternal survival. Transparent documentation of such criteria is essential in similar cases to support ethical decision‐making and guide clinical practice.

Experiences from resource‐limited settings further highlight the challenges in diagnosing and managing cystic lung diseases when advanced biomarkers and imaging modalities are not readily available. For example, Aghajanzadeh et al. reported similar diagnostic and therapeutic challenges in the management of complicated cystic lung disease in northern Iran, where surgical intervention and histopathological evaluation played a central role [12]. Although the underlying pathology may differ from LAM, such reports underscore the importance of adaptable clinical strategies in environments with limited diagnostic resources. These regional experiences, including those from Middle Eastern centers, provide valuable insight into real‐world management approaches and support the relevance of our case in demonstrating multidisciplinary decision‐making under constrained conditions.

Although bilateral pneumothorax and pregnancy‐associated worsening are recognized manifestations of LAM, the present case remains noteworthy because of the particular combination of features at presentation and during management. The patient presented at only 17 weeks of gestation with severe bilateral pneumothorax, near‐complete collapse of both lungs, profound hypoxemia, and acute respiratory distress requiring urgent multidisciplinary intervention. HRCT also demonstrated exceptionally small cysts (< 1 mm), which are atypical relative to the more commonly described cyst size spectrum in LAM. In addition, diagnosis was established in a resource‐limited setting where serum VEGF‐D testing was unavailable, making thoracoscopic biopsy with immunohistochemical confirmation essential. Therefore, the educational contribution of this report is not that pneumothorax or pregnancy exacerbation in LAM has never been described, but that it illustrates an unusually severe early gestational presentation together with diagnostic and therapeutic decision‐making under limited‐resource conditions.

Common symptoms include dyspnea on exertion and recurrent pneumothorax. Less common symptoms include chest pain, dry cough, hemoptysis, ascites, and chylous effusion. Conventional radiographs may show a reticular pattern, multiple pulmonary cysts mimicking honeycomb, and increased lung volume [3, 13]. HRCT typically shows multiple, thin‐walled cysts surrounded by relatively normal lung parenchyma. The size of the cysts can vary, with larger cysts indicating more severe disease. Pneumothorax is common, occurring in over 50% of patients. Pleural effusions can also be present. In some cases, the lungs may appear normal on radiographs despite the presence of pulmonary cysts [3]. Differential diagnoses include other ILDs that present with cystic changes, such as COPD, emphysema, langerhans cell histiocytosis, and lymphoproliferative disorders [14].

Serological tests such as ANA, Anti‐dsDNA, and antiphospholipid antibodies were conducted as part of the differential diagnosis workup to exclude autoimmune or connective tissue diseases that can mimic cystic lung disease, such as systemic lupus erythematosus or lymphocytic interstitial pneumonia [15, 16]. Although these were ultimately normal, their inclusion reflects a comprehensive diagnostic approach. Management with sirolimus, an mTOR inhibitor, is well‐supported by evidence for stabilizing lung function and reducing complications in LAM. The patient was initiated on sirolimus 2 mg/day and continues under regular follow‐up. Sirolimus is generally contraindicated in pregnancy because of potential teratogenic effects, which further justified the medical team′s decision to terminate the pregnancy [17]. Current guidelines recommend avoiding pregnancy while on sirolimus, and therapy duration is typically long‐term, depending on symptom control and lung function stability [18]. Differential diagnoses for diffuse cystic lung disease including emphysema, pulmonary langerhans cell histiocytosis, Birt–Hogg–Dubé syndrome, and connective‐tissue–related ILDs were systematically evaluated. Normal autoimmune serologies and characteristic radiologic findings made autoimmune lung disease less likely. The absence of upper‐lobe–predominant bizarre cysts and nodules argued against langerhans cell histiocytosis. Histopathologic examination demonstrating HMB‐45, desmin, and CD34 positivity provided definitive confirmation of LAM.

This case demonstrates several distinctive features that add to the limited literature on LAM during pregnancy. First, the patient presented with bilateral pneumothorax and near‐complete lung collapse at only 17 weeks of gestation, an unusually severe early presentation. Second, high‐resolution CT revealed exceptionally small cysts (< 1 mm), suggesting a very early radiologic stage or partial visualization due to extensive pneumothorax, which is atypical compared with the commonly described 2 mm to > 5 cm cysts in LAM. Third, management occurred in a resource‐limited setting in which VEGF‐D testing—the recommended biomarker for noninvasive diagnosis was not locally available, necessitating reliance on histopathology for confirmation. These factors underscore the diagnostic and therapeutic challenges faced in low‐resource environments.

A limitation of this report is the absence of serial spirometric data to objectively quantify pulmonary function and treatment response. This was primarily because of the patient′s critical presentation, which precluded baseline testing and limited access to pulmonary function testing during follow‐up. Clinical improvement was therefore assessed using functional status, oxygen requirements, and radiological findings rather than standardized pulmonary function parameters. Another limitation of this report is the lack of detailed longitudinal documentation regarding sirolimus dose titration, serum trough concentrations, and structured adverse‐effect monitoring. Although 2 mg/day was selected as a standard starting dose consistent with prior LAM studies, more complete pharmacologic follow‐up data would strengthen assessment of treatment safety and optimization. Quantitative assessment of the proportion of immunopositive cells was not available from the original pathology report.

## 4. Conclusion

LAM is a rare disease in young women that can present with spontaneous pneumothorax and dyspnea on exertion. Definitive treatment for LAM may require a lung transplant. Patients who are not transplant candidates or who forgo transplant can be managed pharmacologically with sirolimus. Long‐term management includes continued sirolimus therapy at 2 mg/day with regular monitoring for adverse effects and therapeutic response. Pulmonary function testing and high‐resolution CT imaging are planned at 6‐ to 12‐month intervals to assess disease stability. Preventive strategies include avoidance of air travel, smoking cessation, vaccination updates, and consideration of pleurodesis should recurrent pneumothorax occur. The patient will remain under multidisciplinary follow‐up to optimize long‐term outcomes.

## Author Contributions

S. E. and A. A. contributed to the conceptualization, and writing the original draft; M. B., S.E., and A. H. reviewed, supervised and edited the final manuscript.

## Funding

No funding was received for this manuscript.

## Disclosure

All authors have read and approved the final manuscript.

## Consent

Informed consent was obtained from the patient for publication of this case report and accompanying images. A copy of the written consent is available for review by the Editor‐in‐Chief of this journal upon request.

## Conflicts of Interest

The authors declare no conflicts of interests.

## Data Availability

The data that support the findings of this study are available on request from the corresponding author. The data are not publicly available due to privacy or ethical restrictions.
